# Two-body wear of occlusal splint materials from subtractive computer-aided manufacturing and three-dimensional printing

**DOI:** 10.1007/s00784-022-04543-5

**Published:** 2022-05-25

**Authors:** Felix Schmeiser, Uwe Baumert, Bogna Stawarczyk

**Affiliations:** 1 Dental Material Unit, Department of Prosthetic Dentistry, Dental School, University Hospital, LMU Munich, Goethestraße 70, 80336 Munich, Germany; 2 Department of Orthodontics and Dentofacial Orthopedics, University Hospital, LMU Munich, Goethestraße 70, 80336 Munich, Germany

**Keywords:** Computer-aided manufacturing, 3D printing, Two-body wear, Mastication simulation, Occlusal splint materials, Abrasion losses

## Abstract

**Objectives:**

To investigate the two-body wear of occlusal splint materials fabricated from subtractive computer-aided manufacturing (CAM) compared to three-dimensional printing (3DP).

**Material and methods:**

Forty-eight substrates (*n* = 12/material) in the design of a mandibular first molar were fabricated using CAM (CAM-TD, Thermeo, pro3dure medical GmbH, Iserlohn, Germany; CAM-CL, CLEARsplint, Astron Dental Corporation, Lake Zurich, USA) and 3DP (3DP-GI, GR22 flex, pro3dure medical GmbH; 3DP-KY, KeySplint soft, Keystone Industries, Gibbstown, USA). The substrates were subjected to mastication simulation (120,000 cycles, 37 °C, 50 N, 1.3 Hz) opposed to enamel antagonists. The two-body wear was measured through matching of the scanned substrates before and after aging using Gaussian best-fit method. The damage patterns were categorized and evaluated based on microscopic examinations. Data was analyzed using Kolmogorov–Smirnov test followed by 1-way analysis of variance (ANOVA). Pearson correlation was calculated between vertical and volumetric material loss. The failure types were analyzed with Chi^2^-test and Ciba Geigy table.

**Results:**

No difference in two-body wear results between all materials was found (*p* = 0.102). Fatigue substrates showed a perforation for CAM and a fracture for 3DP. No abrasion losses on the antagonists were detected.

**Conclusions:**

3DP substrates showed no differences in two-body wear compared to CAM ones but are more likely to show a fracture. None of the tested materials caused an abrasion on human teeth structure.

**Clinical relevance:**

While therapies with occlusal splint materials are rising, 3DP offers a promising alternative to CAM in terms of production accuracy and therapeutic success at reduced costs.

## Introduction

Occlusal splints have established themselves in numerous areas of dentistry and orthodontics. Their application involves the treatment of disturbed occlusal contacts, temporomandibular jaw malposition/diseases or parafunctions such as grinding and clenching leading to bruxism [1–4]. In addition, for esthetic reasons, they can be applied in orthodontics for tooth regulation, in the form of aligners, or used as transfer splints for adhesive attachment of the brackets in fixed orthodontic splints [5–7].

Occlusal splints are produced using conventional methods involving vacuum injection molding, scattering of acrylic resin [8] or a combination of both [9, 10]. Computer-aided design (CAD) and computer-aided manufacturing (CAM) have automated the manufacturing of occlusal splints using milling technology. As such, manufacturing can be implemented in a fully digital workflow, thus simplifying planning and increasing reproducibility and manufacturing accuracy [11]. On the other hand, higher material consumption, tool wear and associated costs are associated with such a workflow. In this context, three-dimensional printing (3DP) provides a promising and economical alternative to produce occlusal splints [6, 11, 12]. Various methods such as stereolithography (SLA) [13], polyjet and digital light processing (DLP) [14] with layer-by-layer polymerization of resins are available for its fabrication. Related to the reduced material consumption and manufacturing time compared to CAM, multiple areas of occlusal splints are already covered with 3DP [15, 16]. Furthermore, higher accuracy has been observed among others in the fabrication of dental restorations by means of 3DP [11].

Occlusal splint materials are subjected to corresponding loads very similar to the teeth by mastication, whereby different wear mechanisms occur depending on the material and its antagonist [11, 17]. Therefore, materials are expected to behave as close as possible to natural tooth structure in terms of their mechanical properties by opposing teeth or restorations for an appropriate period [11, 17, 18]. Based on clinical investigations, a considerable improvement in discomfort through the nightly use of occlusal splints was observed after 3 months [19], and a reduction of pain of 71.0–72.8% was achieved after 6 months [20]. High accuracy fit as well as low wall thickness of occlusal splints should therefore lead to a maximum therapeutic effect with maximum wearing comfort and enhanced esthetics. Additionally, good abrasion resistance, a low residual monomer content and good stability are required [12, 18, 21, 22].

However, there is no standardization in testing parameters like force, substrates geometry or cycle numbers among others. Additionally, the abrasion resistance of 3DP occlusal splints cannot yet be clearly classified against conventional and milled ones [23]. Forces used in two-body wear investigations vary between 5 and 50 N, with 50 N being used predominantly, which is considered the standard [24]. Besides cylindrical substrates with a flat surface, crown-shaped substrates were used in the investigations representing a more detailed replica of the clinical oral situation [23, 24]. The number of chewing cycles varies according to the different wearing times of occlusal splints, in some cases from 10,000 to 60,000 and up to 200,000 cycles [23]. It should be noted that a higher number of chewing cycles allows for a better differentiation of the values due to the increased abrasion values [24, 25]. Data on the comparison of 3DP occlusal splint materials with conventional and milled ones is very limited [23, 24, 26, 27]. While one investigation reported the highest abrasion losses in printed occlusal splint materials compared to milled and casted products [24], others found no differences in wear resistance [26, 27]. All mentioned investigations used photopolymer resin materials based on dimethacrylate for 3DP. For milled CAD/CAM substrates clear PMMA-based and PC-based fully polymerized blanks were used.

Therefore, the purpose of this in vitro investigation was to evaluate the two-body wear rates of occlusal splint materials from CAM compared to 3DP under clinically relevant conditions. The null hypothesis stated that there is no difference in material losses between all material groups.

## Materials and methods

**Fig. 1:**
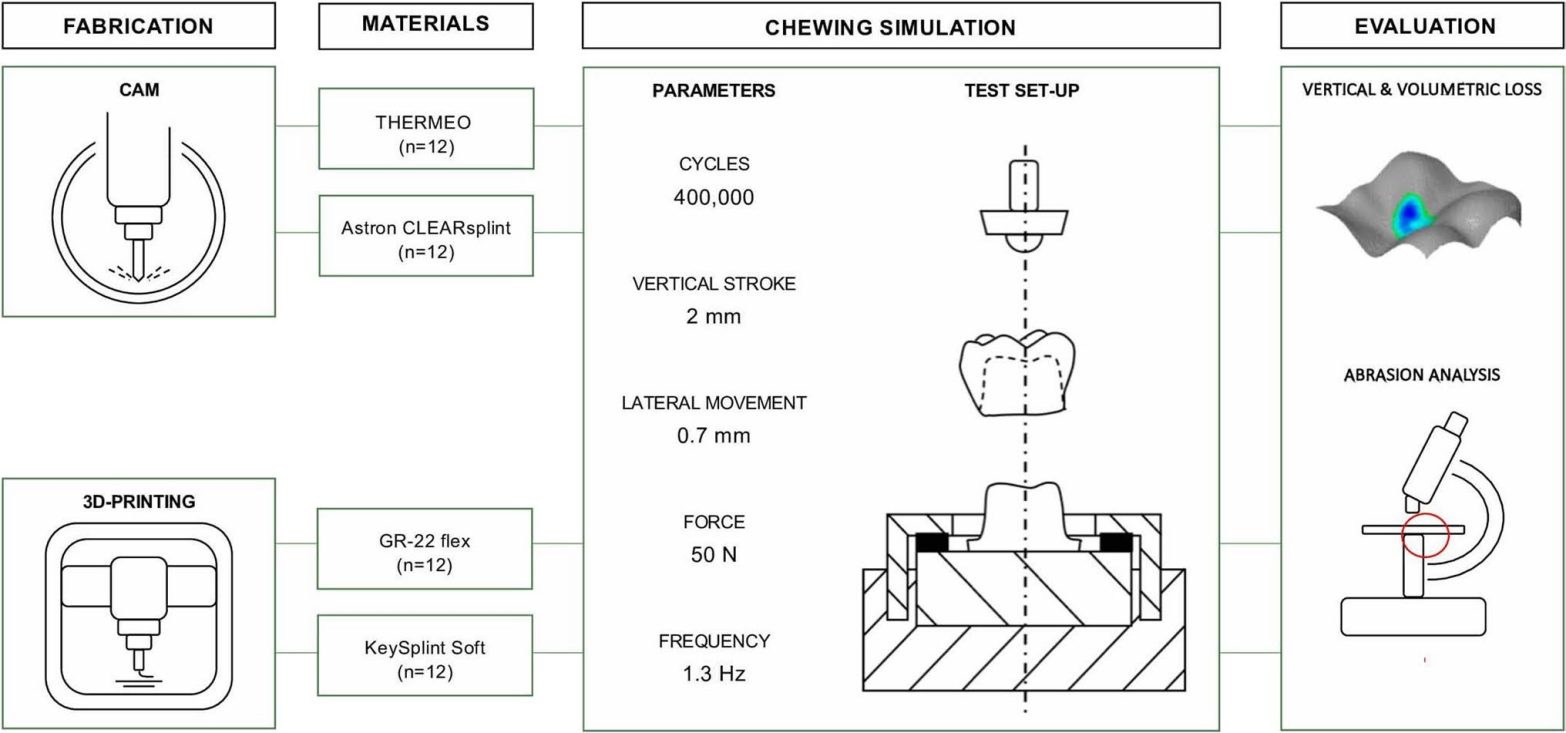
Study design

Substrates (*N* = 48) based on the geometry of a mandibular first molar were fabricated by CAM (*n* = 24, *n* = 12/material) and 3DP (*n* = 24, *n* = 12/material) using the same master standard tessellation language (STL) dataset (Fig. 1). The sample size (*n* = 12 in each group) was selected based on previously published studies [23, 26, 27]. The composition of all utilized materials is reported in Table 1.

**Table 1:** Compositions of the base materials used for the CAM blanks and the photopolymer resin for 3DP based on information provided by the manufacturers (*m*, mass percent; n.g., not given)

Material	Brand	Component	Composition	*m* [%]
CAM-TD	Thermeo	Blank	Methacrylate polymer	< 100
			Dibenzoyl peroxide	< 1
		Liquid	1,2-Cyclohexane dicarboxylic acid diisononyl ester	< 20
			Tetrahydrofurfuryl methacrylate	< 40
			2-Ethoxyethyl methacrylate	< 50
CAM-CL	Astron CLEARsplint	Blank	Methacrylate polymer	75–100
			Dibenzoyl peroxide	0.1–3
		Liquid	2-Ethoxyethyl methacrylate	75–100
3DP-GI	GR-22 Flex	Resin	Oligomers, methacrylic resins, multifunctional	< 75
			Methacrylic resins, monofunctional	> 25
			Photoinitiators (various)	< 2 (in total)
			Pigments/stabilizers	n.g
3DP-KY	KeySplint Soft	Resin	Methacrylate monomer 1	≤ 50
			Methacrylate monomer 2	< 3
			Methacrylate monomer 3	≤ 25
			Photoinitiator 1	< 3

The CAM substrates were generated from blanks made of PMMA (CAM-TD, Thermeo, pro3dure medical GmbH, Iserlohn, Germany; CAM-CL, CLEARsplint, Astron Dental, Lake Zurich, USA). The substrates were digitally nested (Ceramill Mind, Amann-Girrbach, Koblach, Austria) and milled (Ceramill Motion II, Amann-Girrbach) on the blanks (98.5 mm × 20.0 mm). Subsequently, the substrates were cut out of the blank, and the support pins were removed manually.

Analogously, the 3DP substrates were printed either with 3DP-GI (GR22 flex, pro3dure medical GmbH) or with 3DP-KY (KeySplint soft, Keystone Industries, Gibbstown, USA). The 3DP of the substrates (Asiga Max UV, Dr. Stephan Weiß—3DXS, Erfurt, Germany) was coordinated to occur 7 days prior to the mastication simulation. For 3D printing, the substrates were tilted 90° by placing the support structures required for printing on the buccal surface to ensure an accurate reproduction of the occlusal surface (layer thickness ~ 1 µm). Afterwards, the substrates were post-processed by cleaning with isopropyl alcohol (97%) and dried for 30 min at 60 °C. Finally, post curing was proceeded in an inert atmosphere, either with a UV light (CD2, pro3dure medical GmbH) for 2 × 20 min with the 3DP-GI substrate, or by application of 2 × 2000 flashes (Otoflash G171, NK Optik GmbH, Baierbrunn, Germany) to the 3DP-KY substrate.

**Fig. 2:**
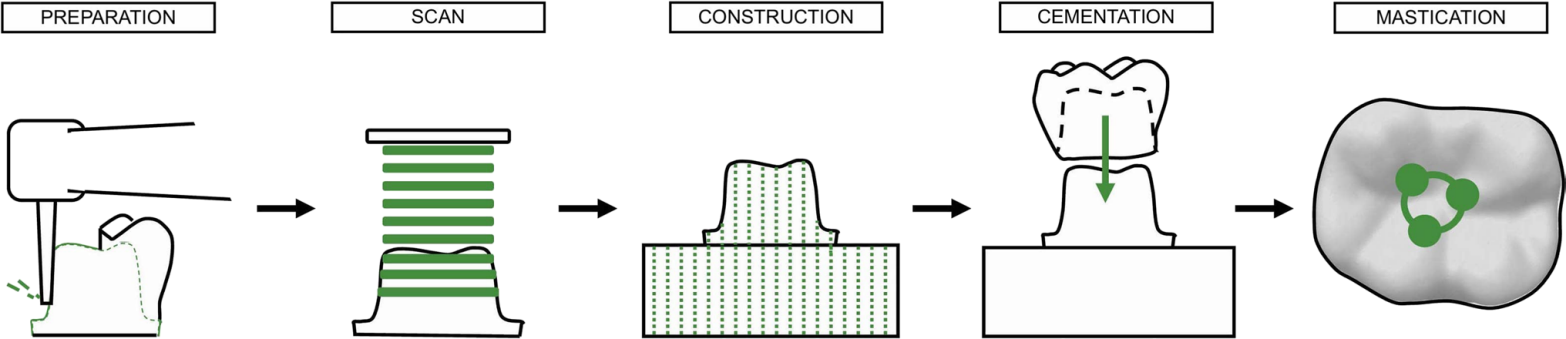
Preparation, development and positioning of the crown-shaped substrate

An abutment for a first lower molar was prepared from a polymer tooth abutment (A-3 Z, frasaco GmbH, Tettnang, Germany) using diamond drills with grainsize of 70 µm and 40 µm following the dental guidelines for preparation of SiO_2_ fixed-dental prosthesis and those made of polymer material [28]. The abutment geometry was digitized using a laser stripe scanner (Ceramill map 400, Amann Girrbach). A cuboid-shaped fixing-base was digitally designed (Meshmixer, ADSK Ireland Ltd., Dublin, Ireland; Ceramill mind, Amann Girrbach) and added to the stump to provide an optimal fixation into the substrate holder of the mastication simulator (Fig. 2).

In each case, the substrates were fixed to the abutment 24 h before initiation of the mastication simulation as follows. Both, the surface of the abutment and the inside of the substrates were blasted pressurized (1.5 bar) with aluminum powder with an average grain size of 110 µm (Korox 110, BEGO Bremer Goldschlägerei, Bremen, Germany). Immediately afterwards, the surfaces were cleaned with compressed oil-free air. Before adhesive bonding (SoloCem, Coltene/Whaledent AG, Altstätten, Schweiz), petrolatum was applied to the stump to provide a slightly flexible bonding according to the manufacturer instructions. The occlusal surfaces of the substrates were sprayed with occlusion spray (ARTI-SPRAY, Dr. Jean Bausch GmbH & Co. KG, Cologne, Germany) for 2–3 s in preparation for the pre-scan (Fig. 1). The surface was digitized with the laser scanner (LAS20, SD Mechatronik, Feldkirchen-Westerham, Germany) operating with a measuring field of 5 mm × 8 mm, a vertical resolution of 0.8 µm and a horizontal resolution of 0.2 µm. Thereafter, the surfaces were ultrasonically cleaned in distilled water (L&R Transistor/Ultrasonic T-14; L&R Ultrasonics, NJ, USA) and air-dried using oil-free air.

Antagonists from enamel were prepared from extracted human maxillary molars donated anonymously by patients in the area of Munich. The ethical harmlessness of the use of human teeth in the in vitro investigation was confirmed through an ethical approval. The human teeth were gently cleaned and stored in a chloramine solution (0.5% Chloramine-T; Sigma-Aldrich Corp, MO, USA; CAS: 149358–73-6) at room temperature (23 °C) over a period of 1 week. Afterwards, the storage solution was replaced by distilled water, and the teeth were stored in the refrigerator at 5 °C. Teeth not older than 6 months were used for antagonist preparation as follows. The mesiobuccal cusp of each mandibular first molar was cut off sectioned out with a separating disc under water cooling. Each cusp was embedded in a cylindrical steel holder using amalgam (Dispersalloy, Dentsply Sirona, Konstanz, Germany). Afterwards, a hemisphere was milled out of each cusp with a stationary drill (BT-BD 120, Einhell Germany AG, Landau an der Isar, Germany) using a hemispherical diamond grinding instrument (*d* = 4 mm).

The substrates and their corresponding opposing enamel antagonists were fixed in the mastication simulator (CS-4, SD Mechatronik GmbH). The crown-shaped substrate was aligned accordingly to the antagonist gaining three contact points, one each with the triangular beads of the cusps (Fig. 2). For the mastication simulation, the following parameters were set: 120,000 mastication cycles at a frequency of 1.3 Hz with 50 N vertical load, a vertical movement of 2 mm and a lateral movement of 0.7 mm. During the simulation, the substrates were constantly wetted with water at a constant temperature of 37 °C.

Following the mastication simulation, the substrates were first evaluated under a digital light microscope at ×20 and ×50 magnification (Keyence VHX-6000, KEYENCE Corporation, Osaka, Japan) to gain a qualitative impression of the abrasion surfaces and then scanned (post-scan) using a laser scanner (LAS20, SD Mechatronik) (Fig. 1). For each scan, the substrate surfaces were again sprayed with occlusion spray (ARTI-SPRAY, Dr. Jean Bausch GmbH & Co. KG) from 20 cm for 2–3 s. Provided that no perforation of the substrates occurred, the digitized surface of pre- and post-scans was evaluated with a matching software (GOM-Inspect 2019, GOM GmbH, Braunschweig, Germany), resulting in a three-dimensional mesh structures with a step size of 0.05 mm, a maximum length of 1.5 mm and a noise of 0.2 mm. The respective 3D structures were then superimposed using the Gaussian best-fit method generating a defined measurement area of the abrasion field. This zone was used as a reference for measuring the volume of the substance removal as a sum integral, as well as the maximum height of the removal. In this context, the vertical loss was defined in the direction of the surface normal to indicate the maximum distance between the surfaces within the selected area. The same methodology was used to control the abrasion losses on the enamel antagonists. However, no microscopic examinations were performed. In between using the antagonists, they were always stored in distilled water in a refrigerator at 5 °C.

Data was analyzed using IBM SPSS Statistics Version 26.0 (IBM Corp., Armonk, NY, USA). Descriptive statistics was reported with mean, standard deviation (SD) and 95% confidence intervals (95%CI). The level of significance was set at *α* = 5%. Since none of the material groups showed deviation of the normality assumption evaluated with the Kolmogorov–Smirnov test, the differences between the wear rates of the material groups were evaluated using one-way analysis of variance (ANOVA) with Scheffé post hoc tests. Pearson correlation was calculated between vertical and volumetric material loss. The failure types were analyzed with *Χ*^2^-test and Ciba Geigy table.

## Results

After mastication simulation, different failure types of the substrates occlusal surfaces were evaluated (Table 2). Substrates containing a perforation or a fracture during the mastication simulation were not included in the statistical analyses of the vertical and volumetric loss values (Table 3).

**Table 2:** Relative frequencies (%) with 95% CI of failure types after mastication simulation

Material	Brand (statistics)	Failure types after mastication simulation		
		**Abrasion**	**Perforation**	**Fracture**
CAM-TD	Thermeo			
	*n* (%)	6 (50)	5 (42)	0 (0)
	95% CI	(14; 73)	(14; 73)	(0; 27)
CAM-CL	Astron CLEARsplint			
	*n* (%)	11 (92)	1 (8)	0 (0)
	95% CI	(60; 100)	(0; 39)	(0; 27)
CAM	Total			
	*n* (%)	17 (71)	6 (25)	0 (0)
3DP-GI	GR-22-flex			
	*n* (%)	4 (33)	(0)	8 (67)
	95% CI	(9; 66)	(0; 27)	(33; 91)
3DP-KY	KeySplint Soft			
	*n* (%)	5 (42)	(0)	7 (58)
	95% CI	(14; 73)	(0; 27)	(26; 85)
3DP	Total			
	*n* (%)	9 (38)	0 (0)	15 (63)

**Table 3:** Descriptive statistics of two-body wear results with mean ± standard deviation (SD) and 95% confidence intervals (CI)

**Material**	***n***	**Vertical loss [mm]**		**Volumetric loss [mm**^**3**^**]**	
		**Mean ± SD**	**(95% CI)**	**Mean ± SD**	**(95% CI)**
*Milled substrates*					
Thermeo	6	− 0.82 ± 0.41	(− 0.54; − 1.09)	− 3.51 ± 1.87	(− 2.31; − 4.70)
Astron CLEARsplint	11	− 0.56 ± 0.25	(− 0.42; − 0.76)	− 2.58 ± 1.34	(− 1.71; − 3.43)
*3D-printed substrates*					
GR-22 flex	4	− 0.45 ± 0.18	(− 0.14; − 0.75)	− 1.76 ± 1.39	(− 0.45; − 3.98)
KeySplint Soft	5	− 0.51 ± 0.09	(− 0.38; − 0.62)	− 1.62 ± 0.67	(− 0.77; − 2.45)

**Fig. 3:**
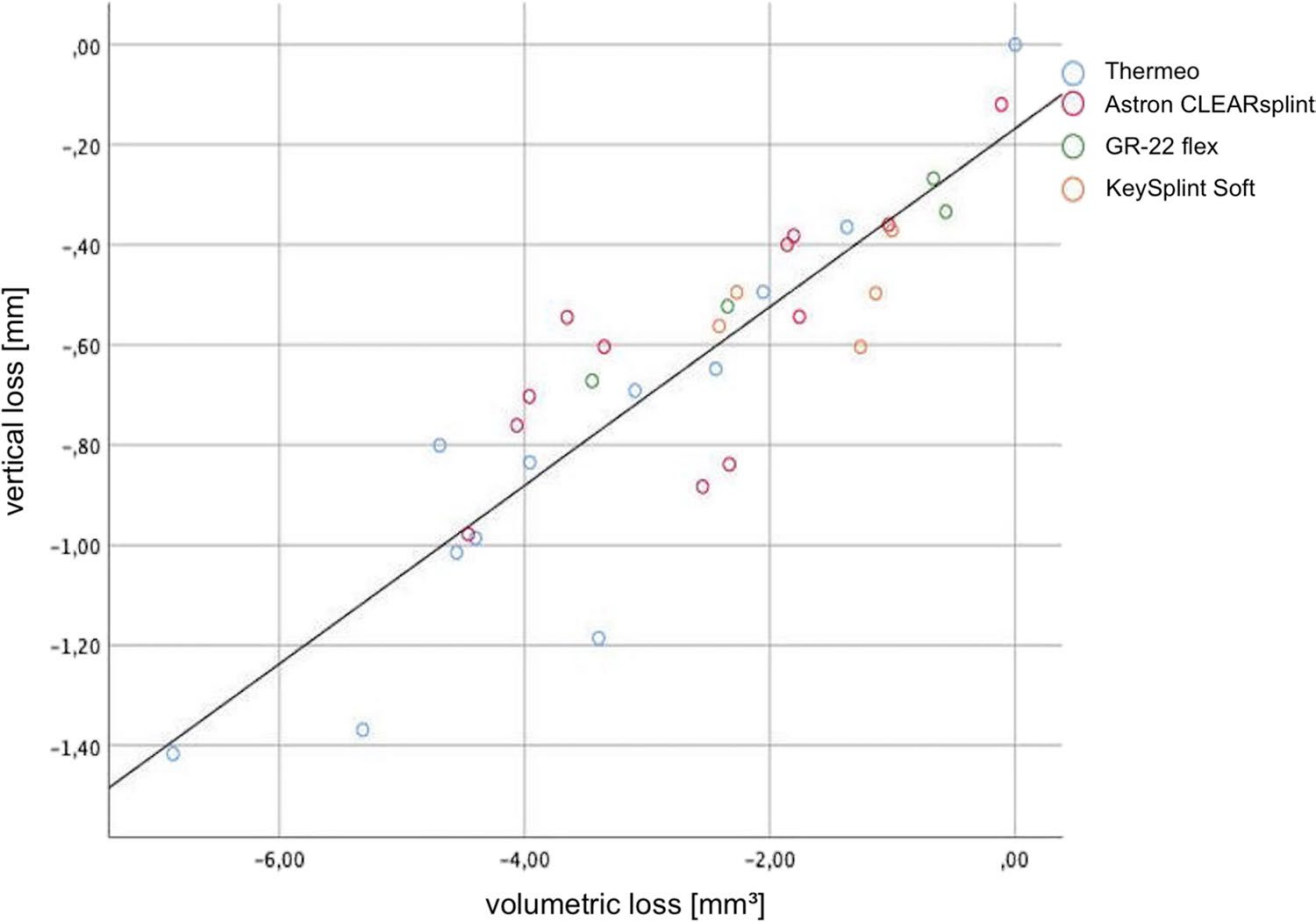
Correlation curve between vertical and volumetric losses (*r* = 0.887, *p* < 0.001)

A positive correlation between vertical and volumetric loss was observed (Pearson correlation; *r* = 0.887, *p* < 0.001, Fig. 3). Consequently, the two-body wear results can be described by only using the vertical material loss. After mastication simulation, the CAM substrates showed more frequent abrasion areas (*Χ*^2^; *p* < 0.001), while the 3DP substrates showed predominantly fracture failure types. Concerning all material groups (CAM-TD: mean − 0.82 mm, 95%CI [− 0.54; − 1.09]; CAM-CL: − 0.56 mm, [− 0.42; − 0.76]; 3DP-GI: − 0.45 mm, [− 0.14; − 0.75]; 3DP-KY: − 0.51 mm, [− 0.39; − 0.62]) no difference in two-body wear results was found (one-way ANOVA; vertical loss: *p* = 0.102) (Table 3). There was no material loss caused by the occlusal splint materials on the enamel antagonists.

**Fig. 4:**
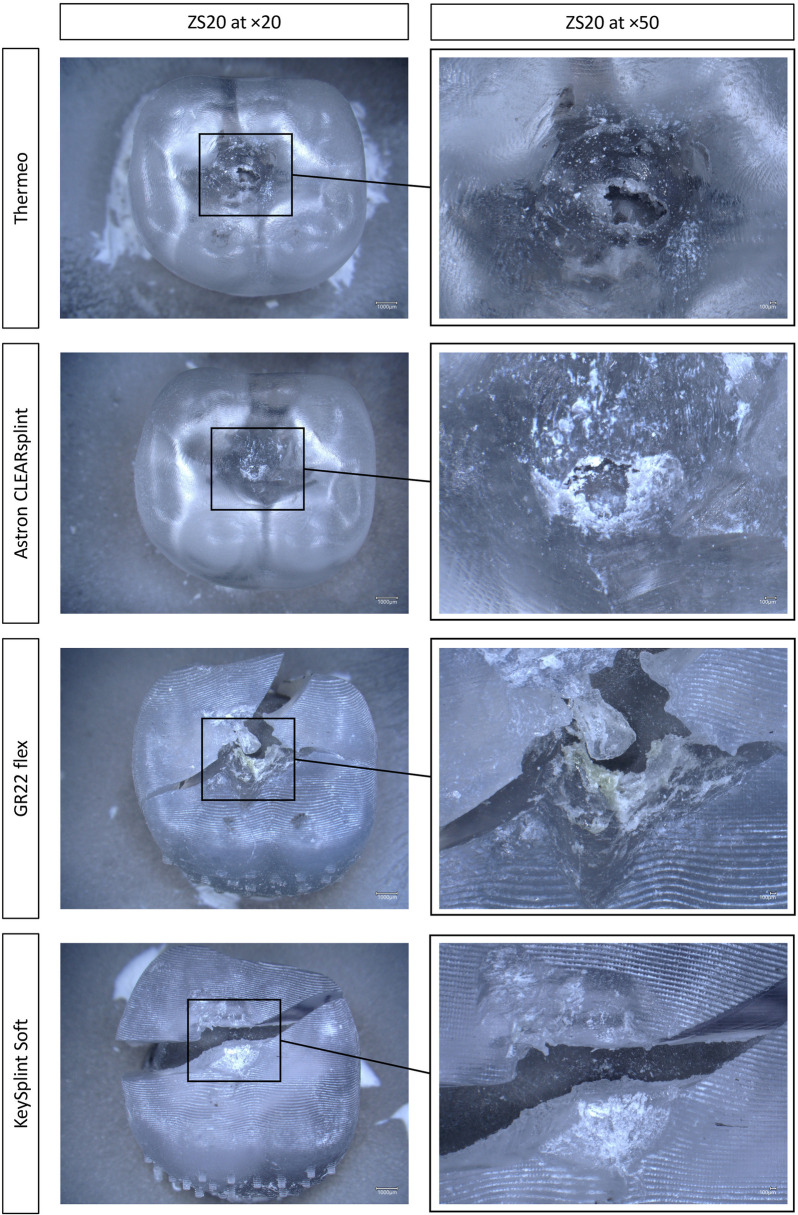
Microscopic images especially of damage patterns from the abrasion surfaces with Keyence VHX 6000

However, differences were found in the distribution of the damage patterns (Table 2) between the individual materials. Typical microscopy images of the observed damage patterns were depicted in Fig. 4. CAM-CL showed predominantly an exclusively abraded surface without perforation or fracture (92%; 95%CI [60;100]). For CAM-TD (50% [14;73]) and 3DP-KY (42% [14;73]), about half of the substrates depicted exclusively abraded surfaces. The same frequency of antagonist perforation through the abrasion surface was observed for the CAM-TD substrate (42% [14;73]). In 3DP-KY, the frequency of fractured substrates was higher (58% [26;85]) than an abrasion-only surface (42% [14; 73]). In comparison to the other substrates, fractures most frequently occurred with the 3DP-GI substrate. Generally, fractures only occurred with 3DP substrates compared to CAM substrates.

Concerning the individual damage patterns, clear differences in the surface finish between the 3DP and CAM substrates were found initially. In the 3DP substrates, grooves caused by the individual layers were visible (arrows in Fig. 4). These structures were ablated at the positions where the force of the antagonist was applied. As expected, the surface of CAM substrates was smoother, but at positions of force application of the antagonist it had a similar effect to that of 3DP substrates.

Substrates with breached surface showed no signs of cracking or fracture in the perforation area, but the marginal areas were clearly roughened. Looking at the fractured substrates, similar structures at the marginal areas were visible, but it was not evident from the damage patterns whether the fracture was preceded by a perforation.

## Discussion

This investigation evaluated the two-body wear of occlusal splint materials under clinically relevant conditions in vitro by means of mastication simulation after 120,000 cycles. In this context, it is evident that the predicted null hypothesis was confirmed, since there was no difference in terms of material loss. The evaluation of the material losses was preceded by a qualitative categorization of the damage patterns. Therefore, it was ensured that only substrates with pure abrasion of the surface were statistically evaluated for the assessment. Substrates in which the surface was breached by the antagonist or in which there was a complete fracture of the substrates were considered separately. The special feature referred to here was the comparison of the results between material groups produced from CAM to 3DP. In addition to the framework conditions of the mastication simulation, antagonists from real teeth were used in order to maintain clinical relevance and realistic reproduction as used in many investigations before [24, 29–32]. The duration of the mastication simulation corresponded to a load of 0.5 years [24], considering more than the average duration of splints of different kinds, which results in a very high load for the material. No thermocycling was applied considering the results of further investigations of the same subject [24, 29, 33]. In comparison with previous investigations, abrasion losses in substrates with different manufacturing methods are seen in the same range [24, 29]. In all groups, as in comparable investigations, there was no abrasion loss on the antagonists made of enamel [24, 29, 34, 35]. No differences between the materials in relation to opposing human teeth were detectable, confirming the results of a further investigation [23]. Therefore, the tested occlusal splint materials do not cause any damage to the human teeth structure.

Based on the results, further conclusions can be drawn by observing the microscopic images. First, no differences can be detected visually within the material groups manufactured by the same method. However, there are clear differences in terms of surface structure between the milled and printed substrates (Fig. 4). Regarding the printed substrates, an undulating structure was formed through the layering process of 3DP. In contrast, the CAM substrates are visually smoother with an even structure. Despite these differences in topography, the abrasion surfaces exhibit a uniformly smooth structure, with no discernible differences. Combined with the similar abrasion values, no influence of the macroscopic surface structure on the abrasion loss results can be assigned. The influence of the surface texture should be determined more precisely by further quantitative investigations.

**Fig. 5:**
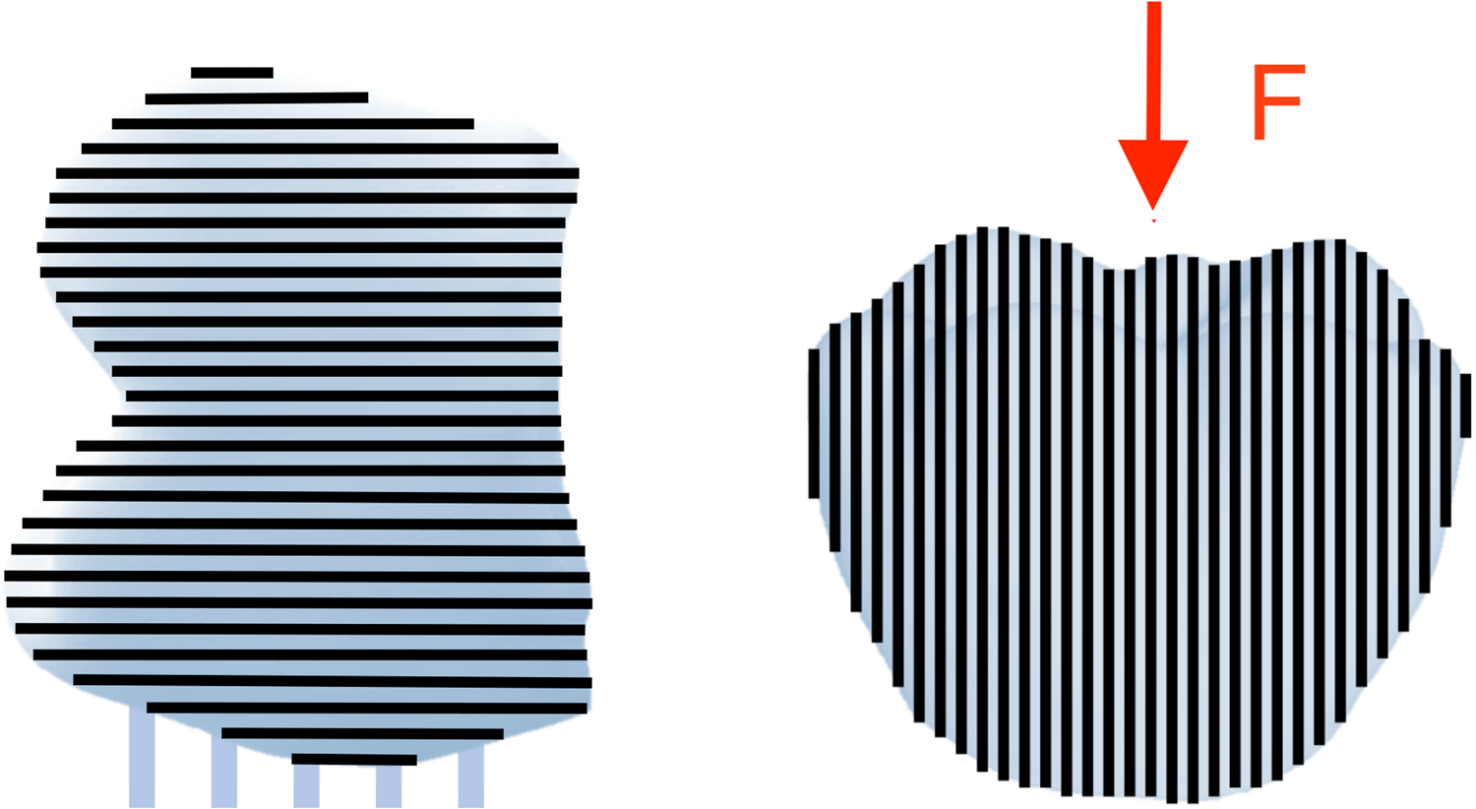
Illustration of the printing direction of the substrates and the respective force direction in the mastication simulator

Differences in the frequency of fractured substrates between the substrates by CAM and 3DP were generally discernible (Table 2). Previous investigations compared the mechanical properties of denture base materials manufactured by different methods showing increased brittleness in 3DP substrates providing lower flexural strength [1, 36, 37]. This is associated with an increased probability of fracture since overloading can be compensated less by deformation. For a more accurate reproduction of the occlusal surfaces, the substrates were tilted by 90° with a horizontal layer orientation (Fig. 5). Accordingly, the layers were oriented parallel to the subsequent loading in the mastication simulator. In addition, the flexural strength properties are also depending on the print direction [37, 38]. In this case, the mechanical properties are reduced as the stability within the printed layer is higher than between the individual layers, leading more likely to a fracture [39]. This influence must be determined more precisely based on further investigations.

Apart from pure abrasion, the substrates from CAM are more likely to show a perforation. Regarding the damage patterns, material residues around the perforation point can be seen in some cases, suggesting the cause of the break-through less because of a fracture and more as a result of the displacement of the material to the marginal areas of the abrasion point. No similar perforation occurred for the substrates from 3DP, although fracture of the substrates was observed. A striking feature was the higher fracture frequency in 3DP-GI compared to 3DP-KY, which can be explained based on the higher flexural strength of 3DP-KY (44–47 MPa) compared to 3DP-GI (10 MPa). However, in case of a potential damage to the occlusal splint material, from a clinical perspective, a fracture is worse than a perforation. In this context, further investigations are necessary to gain more information about the causes of perforations and fractures.

It has been demonstrated that the properties of 3DP materials are largely dependent on the conditions and method of post-processing leading to different results in performance [37, 38, 40, 41]. Related to the post-processing, there were only minor differences between 3DP-GI and 3DP-KY, most probably due to the use of different exposure splints. Currently, there are no investigations that show a correlation between the frequency of fractures and the use of exposure splints with flashing lights or constant exposure of light in a defined period. Additionally, the time between manufacturing and mastication simulation was the same for all material groups. Nevertheless, similar investigations showed a comparatively higher degree of conversion with the Otoflash G171, which might be related to the lower frequency of fractures [37, 38]. In order to be able to make more precise statements about the cause of the differences in the frequency of fracture, further investigations are therefore necessary.

Although this investigation showed that there were no differences in the two-body wear rate within the material groups investigated, the results are limited to the conditions used, since there is no standardized test setup for occlusal splint materials [23, 24, 27]. The two-body wear and fracture behavior could be influenced by the cementation procedure or use of petrolatum resulting in differences to clinical practice which has to be examined in further investigations. Additionally, other materials should be investigated to confirm the influence of the manufacturing method in general. Since extracted teeth were used, differences of the antagonist material such as exposure to bruxism, composition of the tooth or enamel thickness resulting in deviations of the mechanical properties cannot be excluded.

Another limitation of this investigation is that no power analysis was performed a priori to determine an adequate sample size. A post hoc power analysis (R Version 4.1.1 Patched (RStudio 2021.09.2 Build 382), RStudio, Boston, USA) comparing two-body wear values of CAM-TD and 3DP-GI showed that a sample size of 12 specimens per group, an observed effect of − 0.37 mm and a pooled SD of 0.32 mm yielded a statistical power of *p* = 0.8 (80%). It is also necessary to consider that for all materials, apart from CAM-CL, less than 50% of the substrates showed pure abrasion and, accordingly, the number of substrates was low in some cases, leading to a reduced power of the statistical analysis. Nevertheless, the investigations provide further insight into the differences between CAM and 3DP in general and the issue surrounding rail manufacturing. It has been confirmed once again that 3DP can take a firm place in the digitized manufacture of dental products and is thus also an alternative to CAM.

## Conclusions

Within the limitations of this study, the two-body wear of four material groups (two materials each by CAM and 3DP) was carried out at a constant temperature of 37 °C with 120,000 cycles (corresponding to a usage of 0.5 years in vivo) in vitro by means of mastication simulation using antagonists made from enamel. Based on microscopic examination the abrasion areas of the crown-shaped specimens were categorized into three failure types (abrasion, perforation, fracture). The vertical and volumetric material losses of the substrates and their antagonists were determined.

Within the limitation of the present investigation, the following conclusion can be made:

1. No differences were found in terms of material losses between all occlusal splint materials.
2. 3DP occlusal splints showed more frequently a fracture, whereas CAM occlusal splints rather showed a perforation.
3. No material losses on human teeth structure.
